# Cancer extracellular vesicles contribute to stromal heterogeneity by inducing chemokines in cancer-associated fibroblasts

**DOI:** 10.1038/s41388-019-0832-4

**Published:** 2019-05-30

**Authors:** Yutaka Naito, Yusuke Yamamoto, Naoya Sakamoto, Iwao Shimomura, Akiko Kogure, Minami Kumazaki, Akira Yokoi, Masakazu Yashiro, Tohru Kiyono, Kazuyoshi Yanagihara, Ryou-u Takahashi, Kosei Hirakawa, Wataru Yasui, Takahiro Ochiya

**Affiliations:** 1 Division of Molecular and Cellular Medicine, National Cancer Center Research Institute, Tokyo, Japan; 2 Department of Molecular Pathology, Hiroshima University Graduate School of Biomedical and Health Sciences, Hiroshima, Japan; 3 Molecular Oncology and Therapeutics, Osaka City University Graduate School of Medicine, Osaka, Japan; 4 Division of Carcinogenesis and Cancer Prevention, National Cancer Center Research Institute, Tokyo, Japan; 5 Division of Biomarker Discovery, Exploratory Oncology and Clinical Trial Center, National Cancer Center, Chiba, Japan; 6 Department of Cellular and Molecular Biology, Division of Integrated Medical Science, Graduate School of Biomedical Sciences, Hiroshima University, Hiroshima, Japan; 7 Department of Molecular and Cellular Medicine, Institute of Medical Science, Tokyo Medical University, Tokyo, Japan

**Keywords:** Gastric cancer, Cancer microenvironment

## Abstract

Cancer-associated fibroblasts (CAFs), one of the major components of a tumour microenvironment, comprise heterogeneous populations involved in tumour progression. However, it remains obscure how CAF heterogeneity is governed by cancer cells. Here, we show that cancer extracellular vesicles (EVs) induce a series of chemokines in activated fibroblasts and contribute to the formation of the heterogeneity. In a xenograft model of diffuse-type gastric cancer, we showed two distinct fibroblast subpopulations with alpha-smooth muscle actin (α-SMA) expression or chemokine expression. MicroRNAs (miRNAs) profiling of the EVs and the transfection experiment suggested that several miRNAs played a role in the induction of chemokines such as CXCL1 and CXCL8 in fibroblasts, but not for the myofibroblastic differentiation. Clinically, aberrant activation of CXCL1 and CXCL8 in CAFs correlated with poorer survival in gastric cancer patients. Thus, this link between chemokine expression in CAFs and tumour progression may provide novel targets for anticancer therapy.

## Introduction

Cancer-associated fibroblasts (CAFs) are the activated fibroblasts that comprise the major stromal components in the various types of malignancies [1]. CAFs play a pivotal role in cancer development and progression by enhancing cell proliferation, invasion, inflammation and extracellular matrix remodelling [1, 2]. Hence, several reports have documented that CAFs are potential therapeutic targets for cancer treatment [3]. However, CAF heterogeneity comprising subpopulations with distinct phenotypes and functions in tumour progression, has been considered as the major obstacle to investigating the functional role of CAFs and to targeting CAFs in the diagnosis and therapy [4, 5]. The different subpopulations of CAFs exhibit the different gene expression profiles and are likely distinguished by the expression of markers, such as alpha-smooth muscle actin (α-SMA) and pro-inflammatory cytokines [4, 6]. These CAF subpopulations contribute to the chemoresistance of cancer cells and the enhancement of immunosuppression [5, 7, 8]. These recent studies regarding CAF subpopulations provided the concept that an unselective CAF-targeting strategy may leave other CAF subpopulations, leading to cancer progression [4, 5]. Notably, CAF subpopulations seemed to vary according to cancer subtype and aggressiveness [7, 9], suggesting that cancer cells induced the appropriate subpopulation of CAFs for their progression. Thus, the identification of the cancer-derived factors that generate CAF heterogeneity may provide a novel aspect for understanding the tumour microenvironment. Although cell-surface molecules of a certain CAF subpopulation have been reported [5], the molecular mechanisms by which cancer cells govern the CAF heterogeneity and construct an appropriate microenvironment for their metastasis remain unclear.

Growing evidence indicates that cancer cell-derived extracellular vesicles (EVs) serve as regulatory agents in intercellular communications of the tumour microenvironment [10]. EVs contain functional cellular components, such as proteins and microRNAs (miRNAs), and enable the transfer of these principal factors. These components of cancer-derived EVs are functional in the recipient cells and participate in the induction of CAF phenotypes in both proximal surrounding fibroblasts and distal sites [11–13]. Moreover, the specific encapsulated molecules in EVs derived from high-metastatic cancer cells enable the creation of the appropriate tumour microenvironment for cancer metastasis [14–16]. Thus, it is highly plausible that EVs derived from cancer cells with high metastatic capacity contribute to the induction of CAF subpopulations favourable for their metastasis.

Here, with a metastatic model of diffuse-type gastric cancer (DGC), we investigated whether a difference in the fibroblast phenotypes could be observed by comparing between high-metastatic and low-metastatic DGC cells. Our study reveals that cancer cells with high metastatic capacity can generate at least two distinct fibroblast subpopulations: α-SMA-expressing type and chemokine-expressing type. EVs contribute to the formation of a specific subpopulation with chemokine expression. We also evaluated the clinical significance of chemokine-expressing CAFs by immunohistochemistry (IHC) using GC tissue samples. These results provide novel aspects of cancer-derived EVs on the formation of the tumour microenvironment for tumour metastasis.

## Results

### High-metastatic gastric cancer cells strongly influence stromal fibroblast activation

**Fig. 1:**
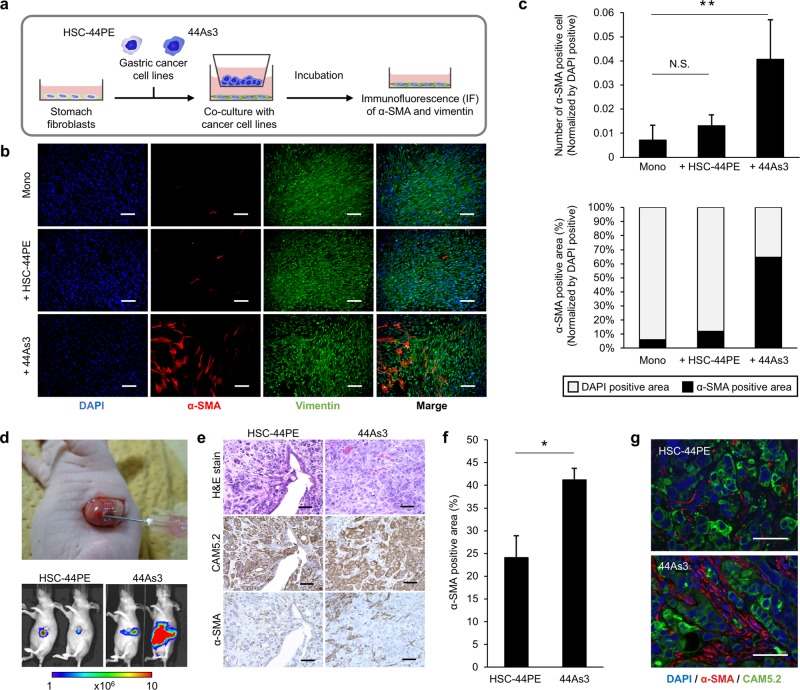
High-metastatic DGC cells strongly induce α-SMA expression in fibroblasts in vivo and in vitro. **a** Schematic protocol of co-culture and immunofluorescence (IF) analysis with α-SMA and vimentin antibody. **b** Representative images of IF. The detection of α-SMA (red), vimentin (green), and DAPI nuclear counterstaining (blue) in iNF-58 mono-culture and co-culture with 44As3 or HSC-44PE. Scale bars, 200 μm. **c** Quantification of the number and area of α-SMA-positive cells in each culture condition. Top: Quantification of α-SMA-expressing cell number normalized to DAPI-positive cell number. Bottom: Quantification of α-SMA-positive area normalized to DAPI-positive area. *n* = 3 biological replicates. Error bars represent the standard deviation (s.d.). **p < 0.01 from one-way ANOVA with Tukey’s honestly significant difference (HSD) test. NS, no significance. **d** Top: Illustrative photograph of an orthotopic mouse model of DGC. Bottom: Representative bioluminescence images of orthotopic mouse models with HSC-44PE or 44As3. By using an IVIS, tumour progression was monitored at 2 weeks after inoculation of DGC cells. **e** Representative microscopic images of HE (top), CAM5.2 (middle), and α-SMA (bottom) staining of primary tumours in the mouse models. Scale bars, 50 μm. **f** Quantification of the α-SMA-positive area. *n* = 3 biological replicates. Error bars represent s.d. *p < 0.05 from Student’s *t*-test. **g** Representative IF images of orthotopic mouse primary tumours of HSC-44PE and 44As3. Scale bars, 50 μm

We previously established two types of DGC cell lines, HSC-44PE, a parental cell line or with low metastatic potential, and 44As3, a high-metastatic cell line [17]. 44As3 was established from HSC-44PE by repeated cycles of orthotopic implantation in nude mice and possessed the characteristics of high dissemination into the peritoneal cavity [18]. We utilized these cell lines to determine whether high-metastatic cancer cells have a strong ability to form a favourable microenvironment for their metastasis. We first cultured immortalized stomach fibroblast lines (iNF-58 and iNF-60) with these DGC cell lines using a transwell culture system (Fig. 1a) and evaluated the fibroblast activation by immunofluorescent staining for α-SMA, a hallmark of myofibroblasts [19]. Vimentin, a typical fibroblastic marker, was positive in all conditions, but the expression of α-SMA increased significantly in the fibroblasts (iNF-58) co-cultured with 44As3 compared with that in other conditions (Fig. 1b, c). Likewise, enhanced α-SMA expression was observed when 44As3 was co-cultured with iNF-60 (Supplementary Fig. S1a). Importantly, only a subset of vimentin-positive fibroblasts expressed α-SMA when co-culture with 44As3 (Fig. 1b), suggesting that not all fibroblasts retained the myofibroblastic phenotype. To further evaluate the difference in fibroblast education capacity between 44As3 and HSC-44PE, these DGC cell lines were orthotopically implanted into nude mice. 44As3 rapidly disseminated into the mouse peritoneal cavity, but the tumour growth of HSC-44PE was restricted to the region where the cells had been implanted (Fig. 1d and Supplementary Fig. S1b, S1c), which was consistent with our previous report [18]. The histopathological analysis showed the active proliferation of CAM5.2-positive cancer cells with extensive interstitial fibrosis in both orthotopic primary tumours (Fig. 1e). However, a marked increase in α-SMA expression in stromal fibroblasts was observed in orthotopic primary tumours of 44As3 but not in those of HSC-44PE (Fig. 1e, f). In addition, we found that the presence of LYVE-1-positive cells (lymphatic vessels) [20], MPO- positive cells neutrophils [21] and CD206-positive cells macrophages [22] was significantly increased in the orthotopic xenograft tumours of 44As3 in comparison to those of HSC-44PE (Supplementary Fig. S2a and S2b). α-SMA-positive stromal cells in orthotopic tumours could be clearly discriminated from cancer cells according to their CAM5.2 expression (Fig. 1g). Since the stromal cells were negatively stained for human-specific mitochondria antibody (Supplementary Fig. S2c) and transplanted DGC expressed luciferase proteins (Supplementary Fig. S2d), they were not derived from implanted cancer cells. We also found that these stromal cells were positive stained for FAP, one of the conventional CAF markers (Supplementary Fig. S2e). These data suggest that high-metastatic DGC cells strongly affect the surrounding stromal fibroblasts and convert them into the activated state, presumably generating the favourable microenvironments.

### High-metastatic DGC cells induce pro-inflammatory genes in the fibroblasts as well as myofibroblast phenotypes

**Fig. 2:**
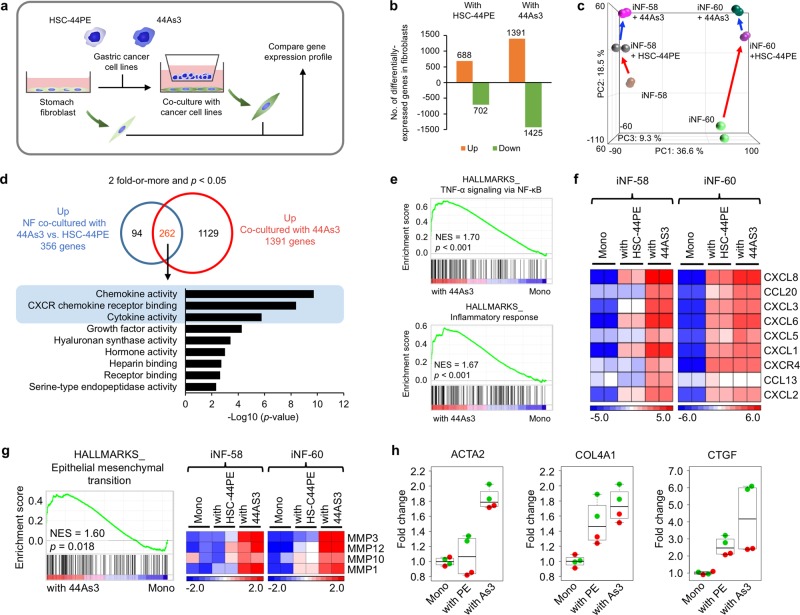
High-metastatic DGC cells induce pro-inflammatory genes and myofibroblast-related genes. **a** Schematic protocol for the gene expression analysis. **b** The number of differentially expressed genes in the fibroblasts co-cultured with HSC-44PE or 44As3 compared with mono-cultured fibroblasts. **c** PCA of gene expression of mono-cultured or co-cultured fibroblasts. **d** GO analysis with 262 selected genes that were significantly up-regulated in the fibroblasts cultured with 44As3. **e** GSEA of the fibroblasts co-cultured with 44As3 versus mono-culture fibroblasts (Mono), highlighting the pro-inflammatory phenotypes. NES: a normalized enrichment score. The *p*-value was calculated by GSEA. **f** A heat map showing CXCL family expression in each culture condition. *n* = 2 technical replicates. **g** Left: GSEA showing the enrichment of epithelial to mesenchymal transition (EMT)-related genes in fibroblasts co-cultured with 44As3. Right: A heat map showing the expression of EMT-related genes in each culture condition. **h** Expressions levels of *ACTA2*, *COL4A1*, and *CTGF* in the gene sets for EMT. Mono-cultured fibroblasts (Mono) and fibroblasts co-cultured with HSC-44PE (with PE) or 44As3 (with As3) are presented. The red dots represent iNF-58 data and the green dots represent iNF60 data

To understand the molecular basis of the fibroblast phenotypes caused by 44As3, we performed whole-transcriptome profiling of fibroblasts in mono-culture and in co-culture with two types of cancer cell lines (Fig. 2a). We found 1391 upregulated genes (> 2-folds, *p* < 0.05) and 1425 downregulated genes (< 0.5-folds, *p* < 0.05) between fibroblasts cultured with 44As3 and mono-cultured fibroblasts (Fig. 2b). Notably, compared to HSC-44PE, 44As3 affected the expression of a larger number of genes in fibroblasts (Fig. 2b). Principal component analysis (PCA) mapping with whole-transcriptome data showed a divergence between iNF-58 and iNF-60, reflecting the difference in their origins (Fig. 2c). This PCA map also revealed a separation of samples into three groups corresponding to the mono-culture and to co-cultures with 44As3 or HSC-44PE (Fig. 2c). These findings suggested that 44As3 and HSC-44PE distinctly affected fibroblast phenotypes in the co-culture system. To further clarify these fibroblast phenotypes, gene ontology (GO) analysis was performed using 262 selected genes whose expression was significantly increased in fibroblasts co-cultured with 44As3 (Fig. 2d and Supplementary Table S1). According to immunohistochemical analysis, the myofibroblastic phenotype with α-SMA was strongly induced in the fibroblasts in the co-culture with 44As3 (Fig. 1b, c); however, the whole-transcriptome analysis indicated the significant alteration of several molecular functions, including the chemokine-related GO terms, “chemokine activity”, “CXCR chemokine receptor binding” and “cytokine activity” (Fig. 2d). Consistent with this finding, gene set enrichment analysis (GSEA) also revealed significant enrichment of gene sets for “TNF-α signalling via NF-κB” and “inflammatory response” in fibroblasts co-cultured with 44As3 versus the mono-culture (Fig. 2e, Supplementary Table S2). The similar pathways were GSEA analysis between the fibroblasts with HSC-44PE and them with 44As3 (Supplementary Fig. S3a–c). Indeed, as shown in the heat map displaying the CXCL family genes among the 262 selected genes, 44As3 significantly enhanced the expression of these chemokine genes in the fibroblasts (Fig. 2f). The changes in the expression of the CXCL family genes in the microarray data were validated by quantitative reverse transcriptase-PCR (qRT-PCR) (Supplementary Fig. S4a). Among these chemokines, the expression of *CXCL8* was remarkably increased in the fibroblasts co-cultured with 44As3 (Fig. 2f and Supplementary Fig. S4a). This observation is consistent with a previous report that CXCL1 and CXCL8 were highly detected in DGC tissues [23]. CAFs secrete various types of chemokines for cancer progression [19], suggesting that 44As3 educates surrounding fibroblasts and forces them to produce chemokines to generate favourable microenvironments. GSEA also revealed that several pathways, including “epithelial mesenchymal transition”, were significantly enriched in fibroblasts co-cultured with 44As3 (Fig. 2g, h, Supplementary Fig. S4b and Supplementary Table S2). Importantly, the gene sets for “epithelial mesenchymal transition” included matrix metalloproteinase (MMP) family genes, the α-SMA gene (*ACTA2*), the type IV collagen gene (*COL4A1*) and the connective tissue growth factor gene (*CTGF*), which were also well recognized as part of the myofibroblast and CAF phenotypes [1]. The expression of *ACTA2* and *CTGF* was also reported to characterize a CAF subpopulation with a myofibroblastic phenotype [4]. Taken together, these findings suggest that 44As3 cell lines strongly induce several activate state in fibroblasts.

### High-metastatic DGC cell line 44As3 generates distinct subpopulations of α-SMA-positive and chemokine-positive fibroblasts

**Fig. 3:**
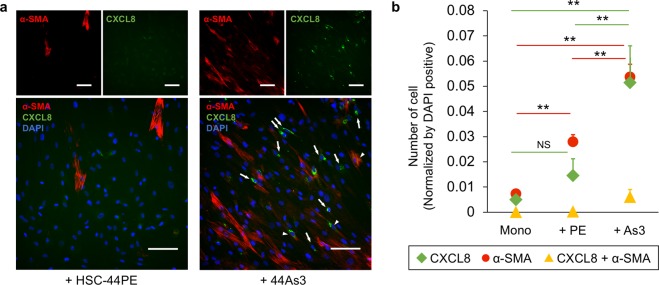
Co-culture with high-metastatic DGC cells generates two distinct subpopulations of fibroblasts. **a** Representative images of IF. The detection of αSMA (red), CXCL8 (green) and DAPI nuclear counterstaining (blue) in fibroblasts after co-culture with 44As3 or HSC-44PE. Arrows: CXCL8-positive fibroblasts. Arrowheads: α-SMA- and CXCL8-double positive fibroblasts. Scale bars, 100 μm. **b** Quantification of the cell numbers in each culture condition. Green: CXCL8-positive fibroblasts. Red: α-SMA-positive fibroblasts. Yellow: α-SMA- and CXCL8-double positive fibroblasts. *n* = 3 biological replicates. *p < 0.05, **p < 0.01 from one-way ANOVA with Tukey’s HSD test

To further characterize the fibroblasts co-cultured with 44As3, we performed immunofluorescence analysis of α-SMA and CXCL8 in the co-culture system and examined the cellular localization of each protein marker. CXCL8 was selected for this experiment because its gene expression was significantly increased in the fibroblasts co-cultured with 44As3. Consistent with the results of the transcriptome analysis, the expression of α-SMA and CXCL8 was strongly induced in iNF-58 with 44As3 as compared with iNF-58 with HSC-44PE (Fig. 3a, b). Cells double positive for α-SMA and CXCL8 were also observed in iNF-58 cells cultured with 44As3 but not in iNF-58 cells cultured with HSC-44PE (Fig. 3a, b). However, interestingly, most of the activated fibroblasts were single positive for either α-SMA or CXCL8 (Fig. 3a, b). This observation is consistent with the previous report that there are two CAF subpopulations, one myofibroblastic and the other pro-inflammatory fibroblasts [4]. The induction of chemokines and α-SMA was also observed in iNF-60 cells cultured with 44As3 (Supplementary Fig. S5a, S5b). These data indicate that co-culture with 44As3 generates their heterogeneity with two distinct subpopulations, α-SMA-positive myofibroblasts and chemokine-positive inflammatory fibroblasts.

### EVs from high-metastatic DGC cells selectively regulated the chemokine expression in the fibroblasts

**Fig. 4:**
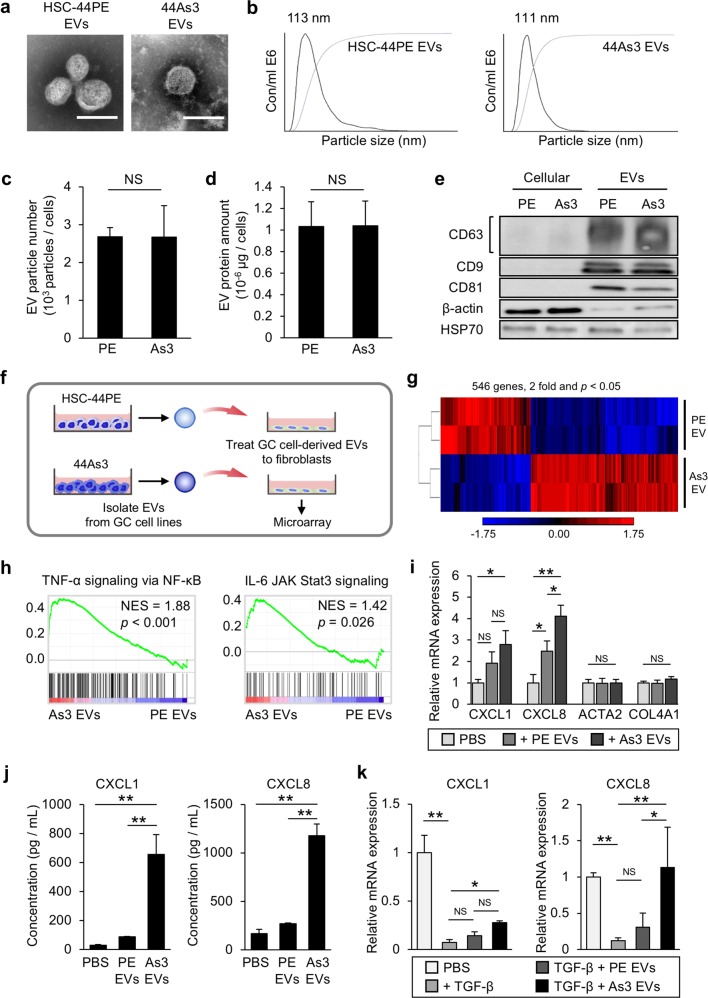
High-metastatic DGC cell-derived EVs induce chemokine expression, but not α-SMA expression in fibroblasts. **a** Representative phase-contrast electron microscopic images of EVs from HSC-44PE and 44As3. Scale bars, 100 nm. **b** Nanoparticle tracking analysis showing the particle size of HSC-44PE EVs and 44As3 EVs. The vertical axis in the graphs shows the number of EV particles (×10^6^)/mL, and the horizontal axis indicates the particle size (nm) of EVs. **c** Comparison of the particle numbers of HSC-44PE EVs (PE) and 44As3 EVs (As3). The vertical axis in the graphs shows the number of EV particles (×10^3^)/cells. *n* = 3 biological replicates. Error bars represent s.d. Student’s *t*-test. NS, no significance. **d** Comparison of the protein amounts in HSC-44PE EVs (PE) and 44As3 EVs (As3). The vertical axis in the graphs shows the amount of EV protein (×10^−6^)/cells. Error bars represent s.d. Student’s *t*-test. NS, no significance. **e** Immunoblot analysis of the conventional EV markers. 500 ng/lane. **f** Schematic protocol for the gene expression analysis in iNF-58 with cancer cell-derived EVs. (**g**) A heat map showing 546 differentially expressed genes (change > 2-fold and *p* < 0.05) in iNF-58 with 44As3 EVs. (*n* = 2) **h** GSEA of iNF-58 with 44As3 EVs versus iNF-58 with HSC-44PE EVs, highlighting the pro-inflammatory phenotypes. **i** The effect of EVs on the expression of *CXCL1*, *CXCL8*, *ACTA2* and *COL4A1* in iNF-58. *n* = 3 biological replicates. Error bars represent s.d. *p < 0.05, **p < 0.01 from one-way ANOVA with Tukey’s HSD test. NS, no significance. **j** ELISA analysis of CXCL1 and CXCL8 secretions in the conditioned medium of iNF-58 with PBS (−) (PBS), HSC-44PE EVs (PE EVs) and 44As3 EVs (As3 EVs). *n* = 4 biological replicates. **p < 0.01 from one-way ANOVA with Tukey HSD test. **k** The effect of EVs on the expression of *CXCL1* and *CXCL8* in iNF-58 cells treated with TGF-β. *n* = 3 biological replicates. Error bars represent s.d. *p < 0.05, **p < 0.01 from one-way ANOVA with Tukey’s HSD test. NS, no significance

We then asked how the heterogeneity of fibroblast phenotypes was formed by 44As3. Growing evidence suggests that EVs are the important cues for understanding the molecular basis underlying the intercellular communications in the tumour microenvironment. We hypothesized that cancer cell-derived EVs conferred the activated fibroblast phenotypes in the co-culture system. To test this hypothesis, we isolated EVs from the conditioned media of HSC-44PE and 44As3 by ultracentrifugation and added them to iNF-58. The EVs used in these experiments were characterized by phase-contrast electron microscopy, nanoparticle tracking analysis and western blotting of conventional EV markers (Fig. 4a–e). To test the EV effect, we applied EVs at 10 μg/mL concentration to the fibroblasts, which is a considerably higher concentration than the one at physiological condition, if all EVs are intact and active after ultracentrifuge. We also confirmed the uptake of these EVs into fibroblasts using PKH-67-labelled EVs (Supplementary Fig. S6a, S6b), and approximately 30% of the fibroblasts were positive for PKH-67 (Supplementary Fig. S6c). To investigate the effect of these cancer cell-derived EVs on the fibroblast phenotype, we next performed microarray analysis and compared the whole-transcriptome profile of EV-treated and non-treated iNF-58 cells (Fig. 4f). We identified 546 genes that were differentially expressed in the fibroblasts between the treatments with HSC-44PE EVs and 44As3 EVs (2-fold and *p* < 0.05, Fig. 4g). To determine the effect of cancer-derived EVs on the fibroblast phenotypes, GSEA was performed for the 44As3 EV-treated iNF-58 and HSC-44PE EV-treated iNF58. As shown in Fig. 4h, the “TNF-α signalling via NF-κB” and “IL-6 JAK Stat3 signalling” pathways were significantly enriched in 44As3 EV-treated iNF-58. The NF-κB and JAK-Stat signalling pathway is one of the major mediators of immune response and inflammatory cytokine expression [24]. As expected, the expression of chemokine family genes such as *CXCL1* and *CXCL8* was significantly increased by 44As3 EV treatment compared with PBS (−) or HSC-44PE EV treatment (Fig. 4i). 44As3 EVs also significantly induced CXCL1 and CXCL8 protein production in the culture supernatant of the fibroblasts (Fig. 4j). Since 44As3 could induce the myofibroblastic phenotype in fibroblasts, we also investigated whether 44As3 EVs induced the expression of α-SMA and collagen type IV genes in iNF-58. Unexpectedly, both of 44As3 EVs and HSC-44PE EVs failed to induce *ACTA2* and *COL4A1* expression in iNF-58 cells (Fig. 4i). The treatment with TGF-β, a potent regulator of the expression of these genes and closely associated with DGC progression [25–27], stimulated *ACTA2* and *COL4A1* expression but suppressed *CXCL1* and *CXCL8* expression in iNF-58 (Fig. 4k and Supplementary Fig. S7a). In contrast, 44As3 EVs restored the expression of these chemokine genes in the presence of TGF-β (Fig. 4k), while HSC-44PE EVs failed. In addition, we also confirmed that when EVs were depleted by the ultracentrifuge method, induction of CXCL8 was significantly decreased (Supplementary Fig. S7b). Since the function of chemokines in cancer proliferation and invasion has been reported [28], we have tested the chemokine effects on the DGC cell line, however there is no significant changes in cell proliferation and invasion (Supplementary Fig. S8a, S8b), implying that these chemokines affect the tumour microenvironment rather cancer cells. These data indicate that 44As3 EVs are key regulators of the generation of chemokine-producing fibroblasts but not of myofibroblasts.

### EVs from high-metastatic DGC cells transferring various miRNAs induce chemokine expression in the fibroblasts

**Fig. 5:**
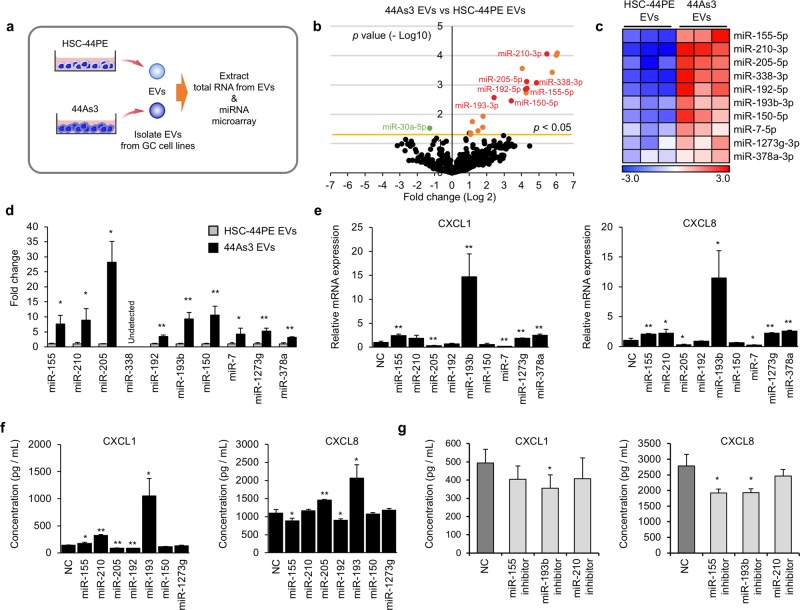
miRNAs in EVs are important for the induction of fibroblast subpopulations. **a** Schematic protocol for the miRNA expression analysis in HSC-44PE EVs and 44As3 EVs. **b** Volcano plot indicating miRNAs differentially expressed between HSC-44PE EVs and 44As3 EVs. Significant differences (change > 2-fold, *p* < 0.05) are indicated in orange (enriched in 44As3 EVs) and green (enriched in HSC-44PE EVs). Representative miRNAs in 44As3 EVs are shown in red. **c** A heat map showing representative miRNAs enriched in 44As3 EVs. **d** qRT-PCR analysis of representative miRNAs in cancer-derived EVs. *n* = 3 biological replicates. Error bars represent s.d. *p < 0.05, **p < 0.01 from Student’s *t*-test. **e** qRT-PCR analysis of *CXCL1* and *CXCL8* expression in iNF-58 cells transfected with representative miRNA mimics. NC: negative control. *n* = 3 biological replicates. Error bars represent s.d. *p < 0.05, **p < 0.01 from Student’s *t*-test. **f** ELISA analysis of CXCL1 and CXCL8 secretions in the conditioned medium of iNF-58 with representative miRNA mimics. NC: negative control. *n* = 3 biological replicates. Error bars represent s.d. *p < 0.05, **p < 0.01 from Student’s *t*-test. **g** ELISA for CXCL1 and CXCL8 after miRNA inhibitions in 44As3 cells in the co-culture system. NC: negative control. *n* = 6 for CXCL1 and *n* = 3 for CXCL8, biological replicate. Error bars represent s.d. *p < 0.05 from Dunnett’s test

We next investigated how 44As3 EVs regulated the chemokine expression in fibroblasts. EVs contain various biomolecules, including proteins and miRNAs, that affect the cellular phenotypes of recipient cells [10, 29]. We performed proteomic analysis of 44As3 EVs and HSC-44PE EVs. Conventional EV marker proteins were detected in both cancer-derived EVs (Supplementary Fig. S9a). Although the amount of some proteins was changed in 44As3 EVs, compared to HSC-44PE EVs (Supplementary Table S3 and S4), we did not find the candidate proteins that were associated with chemokine induction in the fibroblasts (Supplementary Fig. S9b). Importantly, the proteomic analysis indicated that no growth factors, such as TNF-α and TGF-β, were not included in either of the EVs (Supplementary Table S3 and S4). We thus performed miRNA microarray analysis of these cancer-derived EVs (Fig. 5a). There were 756 miRNAs whose signals were detected in the cancer-derived EVs (Supplementary Fig. S9c). Although the PCA mapping of the miRNA profiling showed a rough segregation between HSC-44PE EVs and 44As3-EVs (Supplementary Fig. S9d), only 19 miRNAs were significantly differentially detected in 44As3 EVs compared with HSC-44PE EVs (2-fold and *p* < 0.05, Fig. 5b, Supplementary Table S5). We selected 10 miRNAs from them and performed qRT-PCR to validate the miRNA microarray data (Fig. 5c, d). Among 7 miRNAs validated by qRT-PCR, miR-155 and miR-210 were reported as regulators of pro-inflammatory gene expression [30, 31]. Transfection with miR-155, miR-193b and miR-210 mimics induced CXCL1 and CXCL8 gene expressions and protein production in iNF-58 cells (Fig. 5e, f). To further investigate these miRNA effects on the chemokine induction in the iNF58 cells, we performed the transfection of miR-155, miR-193b and miR-210 inhibitors in the co-culture system with 44As3 (Supplementary Fig. S10a). The knockdown efficiency of each miRNA inhibitor was checked by qRT-PCR (Supplementary Fig S10b). The transfection of these miRNAs reduced the CXCL1 and CXCL8 protein levels in the culture medium (Fig. 5g). Similar results were obtained from qRT-PCR of CXCL1 and CXCL8 in iNF58, although some of them were statistically not significant (Supplementary Fig. S10c). From these data, miR-193b greatly induced the expression of these chemokines and the production of these proteins in iNF-58. Thus, although we could not exclude the possibility that the EV transmission directly influenced gene expression in the cells or other biomolecules expect for miRNAs affected the chemokine expression, our data indicated that some miRNAs in the cancer EVs at least partly contributed to the induction of chemokines in the fibroblasts.

### Chemokine expression in CAFs was closely associated with GC progression

**Fig. 6:**
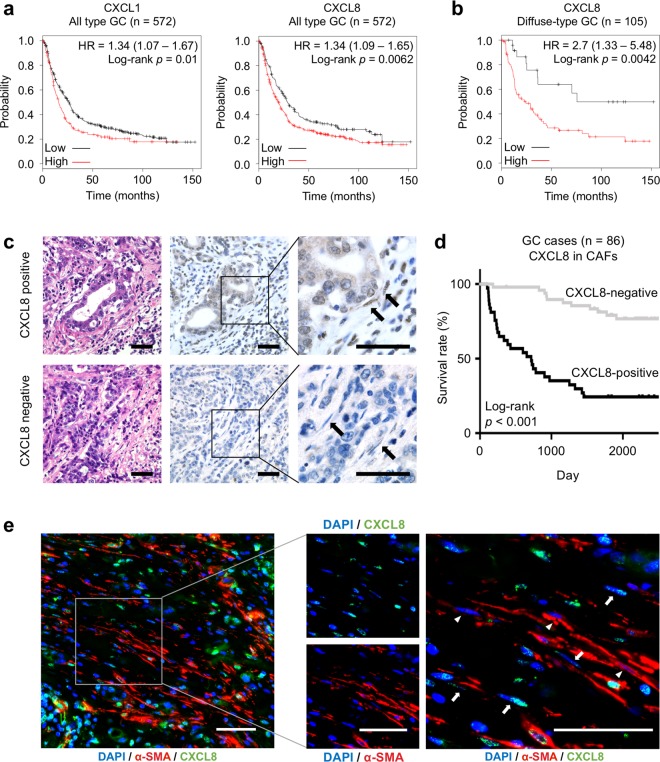
Chemokine expression in the CAF subpopulation was closely associated with GC progression and poor patient prognosis. **a** Kaplan–Meier analysis of the probability of overall survival in GC patients according to the *CXCL1* and *CXCL8* expression. Hazard ratios (HRs) and *p*-values (log-rank test) are shown for each survival analysis. **b** Kaplan–Meier analysis of the probability of overall survival in diffuse-type gastric cancer patients according to the *CXCL8* expression. **c** Representative microscopic images of CXCL8-positive (the presence of CXCL8-positive CAFs) or CXCL8-negative cases in human clinical gastric cancer tissue samples. Arrows: stromal fibroblasts. Scale bars, 50 μm. **d** Kaplan–Meier analysis of the probability of overall survival in 86 gastric cancer patients according to positive (*n* = 37) or negative (*n* = 49) CXCL8 expression in CAFs. **e** Left: representative immunofluorescence images of α-SMA (red), CXCL8 (green), and DAPI nuclear counterstaining (blue) in human gastric cancer. Right: higher magnification indicating the distribution of α-SMA- and CXCL8-positive CAFs. Scale bars, 50 μm. Arrows: CXCL8-single positive CAFs. Arrowheads: α-SMA-single positive CAFs

Finally, we investigated the relationship between chemokine expression in CAFs and the prognosis of patients with GC. Based on the public database, we found that *CXCL1* and *CXCL8* expression was closely associated with poor prognosis in GC patients (Fig. 6a, Supplementary Fig. S11a, S11b). *CXCL8* expression was also correlated with poor patient prognosis even when limited to DGC cases, suggesting that *CXCL8* expression is important for the progression of DGC (Fig. 6b). We also confirmed the correlation of ACTA2 expression and patient prognosis (Supplementary Fig. S11c). These data suggested that both chemokine and α-SMA expression were important to GC progression. To further examine the relationship between CXCL8 expression in CAFs and patient prognosis, we performed IHC using 86 GC tissue samples, including 40 diffuse-type GC cases. In the GC tissue samples, CXCL8 was detected in stromal fibroblasts as well as in malignant epithelial cells and inflammatory cells (Fig. 6c, Supplementary Fig. S12a). We then evaluated the CXCL8-positive and CXCL8-negative cases by counting CXCL8-positive stromal fibroblasts and tested the correlation with the clinicopathological parameters. CXCL8-positive cases were positively correlated with gender (*p* = 0.0346), T grade (depth of tumour invasion: *p* < 0.0001), N grade (degree of lymph node metastasis: *p* < 0.0001), M grade (degree of distant metastasis: *p* *=* 0.0187) and tumour stage (*p* < 0.0001) (Table 1). Histologically, CXCL8 expression in the stromal fibroblasts was more frequently observed in the DGC cases (*p* < 0.0001, Table 1). Consistent with the public database, analysis of the CXCL8 staining in CAFs revealed that CXCL8 positivity in CAFs was significantly associated with poor prognosis in GC patients (log-rank *p* < 0.001, Fig. 6d). Although we also found a correlation between CXCL8 expression in the malignant epithelial cells and poor patient prognosis (log-rank *p* = 0.024, Supplementary Fig. S12b), CXCL8 expression in CAFs was more likely to be associated with poor patient prognosis (log-rank *p* = 0.011, Supplementary Fig. S12c). In addition, expression of α-SMA in CAFs were also associated with poor prognosis in GC patients (log-rank *p* < 0.001, Supplementary Fig. S12d). Notably, at least in part, CXCL8 and α-SMA positivity were observed in the different fibroblasts, displaying CAF heterogeneity in human GC cases (Fig. 6e, arrow and arrow head), which was consistent with the in vitro experimental results. Similar result was also obtained from IHC analysis of CXCL8 with FAP, one of the conventional CAF markers (Supplementary Fig. S13).

**Table 1:** Association between CXCL8 expression in CAFs and clinicopathologic parameters in 86 GC patients

Clinicopathological parameters	CXCL8 expression in CAFs		*p*-value
	Negative	Positive	
Age			
<65	16	11	*p* *=* 0.8181
≥65	33	26	
Gender			
Male	29	30	*p* *=* 0.0364
Female	20	7	
pT grade			
T1,2	30	5	*p* *<* 0.0001
T3,4	19	32	
pN grade			
N0	38	7	*p* *<* 0.0001
N1-3	11	30	
pM grade			
M0	48	30	*p* *=* 0.0187
M1	1	7	
pStage			
I	28	5	*p* *<* 0.0001
II–IV	21	32	
Histology			
Intestinal	37	9	*p* *<* 0.0001
Diffuse	12	28	
Total	49	37	

## Discussion

The diversity of cell-of-origin and nonuniformity of the protein markers in CAFs have been considered to contribute to their heterogeneity and make it difficult to study the nature of CAFs [6, 32]. It has been suggested that the conventional CAF markers, such as α-SMA, FAP and PDGFR-β, are incompetent to distinguish the specific subpopulations of CAFs individually. The recent promising findings regarding the distinct functions of the CAF subpopulations provide novel approaches for the biological function and targeting of CAFs [4, 5, 7, 8, 33]. Several studies have reported that a number of factors could reprogram the potential origin cells into CAFs. For instance, TGF-β and SDF-1 signalling pathways are major regulators of CAF phenotypes [34]. Additionally, the role of PDGFs in the recruitment and functionality of CAFs is well known [9, 35]. However, how cancer-derived factors governed the functional heterogeneity of CAFs and formed the appropriate tumour microenvironment remained obscure. In the present study, we identified a hitherto unrecognized factor to generate a specific fibroblast phenotype within the tumour microenvironment. EVs derived from high-metastatic DGC cells selectively enhanced the expression and production of chemokines in fibroblasts. Intriguingly, only a few miRNAs, including miR-155, miR-210 and miR-193b, were different between 44As3 EVs and HSC-44PE EVs, suggesting this small number of miRNAs was enough to induce of the chemokine-expressing phenotype. In this study, we didn’t evaluate these miRNA levels in patient serum; if there is a correlation between serum miRNA levels and stromal chemokines, detection of the miRNAs in serum might be useful for the diagnosis of gastric cancer as a liquid biopsy. In contrast, myofibroblastic phenotypes, such as α-SMA and COL4A1 expression, were induced by TGF-β treatment in our model, as conventionally thought. We also observed the heterogeneous distribution of CXCL8 and α-SMA in CAFs within human GC tissues. These findings suggest that the forced induction of chemokine-expressing phenotype by high-metastatic DGC cell-derived EVs leads to the formation of the functional heterogeneity of CAFs within the tumour microenvironment (Supplementary Fig. S14). Importantly, only high-metastatic cancer cells possessed such potential, implying that highly malignant cancer cells force the surrounding fibroblasts to generate appropriate CAF subpopulations for their progression and metastasis.

A number of studies have indicated the functional role of chemokines, including CXCL1 and CXCL8, in the tumour microenvironment [28]. Overexpression of CXCL1 and CXCL8 in cancer cells correlated with poor prognosis in GC patients [36, 37]. The forced expression of CXCL8 in GC cell lines promoted their progression through constructing a favourable microenvironment, such as by promoting angiogenesis [38]. Cancer cell-derived CXCL1 mediates the recruitment of bone-marrow mesenchymal cells (BM-MCs), one of the origin cells of CAFs, into the tumour microenvironment [39]. In addition to these important roles of cancer cell-derived chemokines in cancer progression, our study demonstrated that CXCL8 in CAFs was closely associated with GC progression and worse prognosis of GC patients. Likewise, *CXCL1* expression increased upon treatment with 44As3 EVs and correlated with poor patient prognosis in the public database analysis. Although we could not evaluate all of the chemokines in the fibroblasts that were educated by high-metastatic DGC cells, our findings suggest that chemokines secreted from the CAF subpopulation play an essential role in the progression of GC. However, the precise mechanisms by which chemokines from CAF subpopulations affect GC metastasis remains unknown. Chemokines contribute not only to promoting the tumour growth and invasion but also to stimulating the growth and migration of endothelial cells, neutrophils and macrophages [28]. Given that the presence of lymphatic endothelial cells, neutrophils and macrophages increased in the tumours formed by high-metastatic DGC cells in the mouse xenograft, it is possible that chemokines from the CAF subpopulation contribute to the recruitment of these cell types into the tumour microenvironment. Since rodents lack the CXCL8 gene [40], the functions of CAF-derived chemokines could not be identical between xenograft tumours and clinical GC tissues. However, when we look at our gene expression microarray data, it clearly indicated that a number of chemokines such as CCL20, CXCL3, CXCL6, CXCL1 and CXCL2, were also up-regulated in the co-culture with 44As3. For example, it has been reported that CXCL1, a functional homologue of CXCL8 in mice, recruits CD11b^+^ Gr1^+^ myeloid cells in vivo, which contributes to the cancer progression [41]. CXCL1, CXCL2 and CXCL3, known as the ligands for CXCR2, are potent chemoattractant of neutrophils [42, 43]. CXCL6 contributed to the recruitment of neutrophils within the tumour microenvironment [21]. In particular, the neutralization antibody of CXCL6 attenuated the chemotaxis of neutrophils and inhibited tumour growth and metastasis in mouse model [44]. Therefore, our findings and previous studies collectively imply that the chemokines from the CAF subpopulation orchestrate the appropriate tumour microenvironment for cancer progression and metastasis.

Our data from human IHC revealed at least two types of CAF subpopulations, including the myofibroblastic phenotype and chemokine-expressing phenotype in DGC, and our study also found additional CAF subpopulations, such as cells double positive or double negative for α-SMA and CXCL8 (Fig. 3 and Fig. 6e). Given that TGF-β and EVs exerted the opposite effects on the fibroblast phenotypes, the results suggest that the competition of EV-dependent and EV-independent mechanisms may result in the induction of CAFs with the expression of both genes. However, because our study was limited to an in vitro transwell co-culture system, other mechanisms that are independent of humoral factors may exist. It has been reported that the direct interaction with cancer cells or the extracellular matrix is important for the induction and maintenance of CAF phenotypes [45]. Two studies regarding the CAF subpopulations in breast cancer and ovarian cancer indicated that four subsets can be distinguished, one of which is enriched in close proximity of cancer cells [7, 8]. Furthermore, in pancreatic ductal adenocarcinoma (PDAC), the α-SMA-positive CAF subpopulation was regulated by juxtacrine interactions [4]. These studies showed that the direct interaction of cancer cells and fibroblasts led to the induction of CAF subpopulations with a myofibroblastic phenotype. However, our DGC cell model showed that the α-SMA-positive CAF subpopulation appeared without juxtacrine interactions. A simple explanation for this difference in CAF induction could be the difference in tumour cell types and the tissue-specificity of fibroblasts among tumours. Moreover, while CAF subpopulations with a myofibroblastic phenotype may have a potent capacity for cancer progression in breast and ovarian cancer, the CAF subpopulation without a myofibroblastic phenotype was also important for the progression of PDAC. Consistent with the PDAC study, we also showed that the chemokine-expressing CAF subpopulation was strongly associated with poor prognosis in GC patients. Collectively, it is possible that the molecular mechanisms governing CAF subpopulations may be differ among organs. Further examination should be performed to complete clarify the molecular mechanisms and function of CAF heterogeneity across the tissues.

Although this evidence highlighted the important roles of miRNAs in EVs from highly metastatic gastric cancer cells in creating tumour microenvironment, there are a few criticisms of the present study. One of these issues is that it is unclear if the fibroblasts which received EVs directly differentiate into the chemokine-expressing fibroblasts. Based on our in vitro experiment data, when fibroblasts were treated with PHK-67-positive EVs, we observed approximately 30% of the cells positive with PKH-67. Thus, since the percentage of CXCL8 positive fibroblasts is approximately 3%, not all cells which received EVs could differentiate into inflammatory fibroblasts. Also, we could not show the CAF heterogeneity by IHC when 44As3 EV were treated. It is likely due to the limitation of the detection of secreted protein in the cells, or other secretion factors cooperatively functions to induce more chemokines. In addition, we could not show the direct evidence that miRNAs in EVs mediate the inflammatory CAF subtypes. Although it is technically difficult to prove completely because miRNA inhibition might influence cancer cell phenotypes, further investigation should be required.

Another limitation is that there is no direct evidence of the function of cancer EVs in in vivo situation. In other words, we could not show the EV transfer in vivo, although other research group demonstrated the in vivo EV transfer using Cre-loxP system [46]. According to our in vitro experiments, it is clear that cancer EVs have the ability of educating stromal cell, inducing chemokine expression selectively. Due to the technical difficulty, such as the amount EVs and exposure period of EVs, of the experiment to examine the effect of cancer EVs on the tumour progression, our data didn’t show the direct evidence that cancer EVs created the tumour microenvironment and promoted the progression and metastasis of gastric cancer in vivo. To answer these critical questions, a novel experimental model, which can accurately and readily detect the EV uptake in vivo, is needed.

Several reports have demonstrated that EVs derived from cancer cells can convert the potential origin cells into CAF phenotypes. For example, chronic lymphocytic leukaemia cells secrete proteins and miRNAs via EVs and confer the CAF-like phenotypes on the endothelial cells and mesenchymal stem cells [47]. Fibroblast motility, one of the properties of CAFs, is promoted by miR-9 in EVs from breast cancer cell lines [48]. The delivery of hepatocellular carcinoma cell-derived miR-1247 via EVs promotes chemokine expression in fibroblast within the lung pre-metastatic niche [13]. Despite these promising findings, however, no study has focused on the potential of EVs that induce a specific phenotype of activated fibroblasts and contribute to the formation of CAF heterogeneity. In addition, these studies did not evaluate the difference in the functional roles of EVs and other humoural factors for the induction of CAFs. In this study, we demonstrated the important role of EVs in CAF subpopulations and found that the effect of EVs on the induction of CAF-like phenotypes could be clearly distinguished from that of TGF-β. TGF-β suppressed chemokine expression in fibroblasts, but high-metastatic cancer cell-derived EVs recovered chemokine expression. The failure of EVs derived from low-metastatic DGC cells to induce chemokines under TGF-β treatment indicates that EVs derived from high-metastatic cancer cells have a potent capacity to generate the fibroblast subpopulation with chemokine expression. We also identified several EV miRNAs, such as miR-155, miR-210 and miR-193b, which significantly induced chemokine expression in the fibroblasts. Nevertheless, although it is evident that EVs and humoral factors including TGF-β could affect all fibroblasts, the precise mechanisms by which these miRNAs in EVs regulate CAF subpopulations remain unclear. We envision a potential explanation for this observation. Fibroblasts with the capacity of differentiating certain CAF subpopulations may pre-existed in tumour microenvironment. In other words, CAF heterogeneity might be primarily depended on the property of stromal cells, such as epigenetic status.

## Materials and methods

### Tissue samples

For IHC analysis of CXCL8, formalin fixed paraffin embedded (FFPE) tissue from 86 GC patients including 40 diffuse-type and 46 intestinal-type were collected. The samples were randomly selected from patients who underwent surgery at Hiroshima University Hospital or an affiliated hospital in the years 2005–2008. Comprehensive approvals for basic or clinical research were obtained from all of the patients. This study was conducted in accordance with the Ethical Guidance for Human Genome/Gene Research of the Japanese Government and Human Genome Research of Hiroshima University. Pathological diagnosis was based on the 14^th^ edition of Japanese Classification of Gastric Carcinoma and the 4^th^ edition of the WHO Classification of Tumours of the Digestive System.

### Statistical analysis

Values are represented as the mean ± s.d. for technical replicates. Statistical analyses between two groups were performed using a Student’s *t*-test. One-way analysis of variance (ANOVA) was used to determine significant differences among three groups, followed by Tukey honestly significant difference (HSD) post hoc comparisons. A *p*-value less than 0.05 was considered statistically significant.

More detailed version of methods and additional methodology are included in Supplementary Methods.

## Supplementary information

Supplementary Figure 1

Supplementary Figure 2

Supplementary Figure 3

Supplementary Figure 4

Supplementary Figure 5

Supplementary Figure 6

Supplementary Figure 7

Supplementary Figure 8

Supplementary Figure 9

Supplementary Figure 10

Supplementary Figure 11

Supplementary Figure 12

Supplementary Figure 13

Supplementary Figure 14

Supplementary Table 1

Supplementary Table 2

Supplementary Table 3

Supplementary Table 4

Supplementary Table 5

Supplementary Table 6

Supplementary Information
