# Supplementary material for: Cancer extracellular vesicles contribute to stromal heterogeneity by inducing chemokines in cancer-associated fibroblasts
Source: Oncogene. 2019 May 30;38(28):5566–79. doi: 10.1038/s41388-019-0832-4 (PMC6755971; doi:10.1038/s41388-019-0832-4)
Supplement: Supplementary file 21 — Supplementary Information [file 41388_2019_832_MOESM21_ESM.pdf]

Supplementary information

Cancer extracellular vesicles contribute to stromal heterogeneity by inducing chemokines in cancer-associated fibroblasts

Yutaka Naito[1], Yusuke Yamamoto[1], Naoya Sakamoto[2], Iwao Shimomura[1], Akiko Kogure[1], Minami Kumazaki[1], Akira Yokoi[1], Masakazu Yashiro[3], Tohru Kiyono[4], Kazuyoshi Yanagihara[5] Ryou-u Takahashi[1, 6], Kosei Hirakawa[3], Wataru Yasui[2] and Takahiro Ochiya[1, 6*]

1 Division of Molecular and Cellular Medicine, National Cancer Center Research Institute.

2 Department of Molecular Pathology, Hiroshima University Graduate School of Biomedical and Health Sciences.

3 Molecular Oncology and Therapeutics, Osaka City University Graduate School of Medicine.

4 Division of Carcinogenesis and Cancer Prevention, National Cancer Center Research Institute.

Division of Biomarker Discovery, Exploratory Oncology and Clinical Trial Center, National Cancer Center.

6 Department of Cellular and Molecular Biology, Division of Integrated Medical Science, Graduate School of Biomedical Sciences, Hiroshima University.

7 Department of Molecular and Cellular Medicine, Institute of Medical Science, Tokyo Medical University.

*Corresponding Author

Supplementary legends for Figure S1-S14

Supplementary Methods

Supplementary References

Supplementary legends

Supplementary Figure S1. α-SMA expression in iNF-60 cultured with GC cell lines, and in vivo imaging in mouse xenografts. (a) Representative images of IF. Phase contrast images and the detection of α-SMA (red) and DAPI nuclear counter staining (blue) in iNF-60 mono-culture, co-culture with 44As3 or HSC-44PE. Scale bars, 200 μm. **(b)** Representative images of 44As3 and HSC-44PE transplants. **(c)** To show the fold change, luminescence data of 4 weeks after transplantation of each cell line were normalized with the data of day 3. $p < 0.05$, Student's t-test.

Supplementary Figure S2. Immunohistochemistry analysis of LYVE-1, MPO, CD206, and the markers for detection of cancer cells and CAFs. (a) Representative microscopic images of LYVE-1, MPO, and CD206 staining of primary tumours in orthotopic mouse models. Top: the primary tumour in orthotopic mice with HSC-44PE. Middle: the primary tumour in orthotopic mice with 44As3. Bottom: higher magnification of the primary tumour in orthotopic mice with 44As3. Scale bars, 50 μm. **(b)** Quantification of the number and area of LYVE-1-, MPO- and CD206- positive cells in each primary tumour. $n = 3$ biological replicates. Error bars represent s.d. $*p < 0.05$ from Student's *t*-test. **(c)** Representative microscopic images of Human mitochondria staining of primary tumors in the mouse with 44As3. Scale bars, 50 μm. **(d)** Representative microscopic images of mouse primary tumour stained with luciferase antibody. Scale bars, 50 μm. **(e)** Representative microscopic images of FAP staining of the primary tumour in orthotopic mouse with 44As3. Scale bars, 50 μm. Arrows: FAP-positive stromal cells.

Supplementary Figure S3. The comparison of transcriptome profile between the fibroblasts with HSC-44PE and them with 44As3. (a) GSEA of the fibroblasts with 44As3 (with 44As3) vs the fibroblasts with HSC-44PE (with PE), highlighting the pro-inflammatory phenotypes. NES: a normalized enrichment score. The *p*-value was calculated by GSEA. **(b)** GSEA of the fibroblasts with 44As3 (with 44As3) vs the fibroblasts with HSC-44PE (with PE), highlighting epithelial to mesenchymal transition (EMT). **(c)** GSEA showing the enrichment pathways in fibroblasts co-cultured with 44As3 compared with the fibroblasts co-cultured with HSC-44PE.

Supplementary Figure S4. qRT-PCR analysis of chemokines and EMT-related genes in the fibroblasts cultured with GC cells. (a) The expression levels of chemokine genes in iNF-58 cells. Mono-cultured fibroblasts (Mono) and fibroblasts co-cultured with HSC-44PE (+ PE) or 44As3 (+ As3) are presented. $n = 2$ biological replicates; $n = 3$ technical replicates. Error bars represent s.d. $^{*}p < 0.05$, $^{**}p < 0.01$ from one-way ANOVA with Tukey HSD test. NS, no significance. **(b)** The expression levels of *MMP1*, *MMP3*, *MMP10*, and *MMP12* in iNF-58 cells. Mono-cultured fibroblasts (Mono) and fibroblasts co-cultured with HSC-44PE (+ PE) or 44As3 (+ As3) are presented. $n = 2$ biological replicates; $n = 3$ technical replicates. Error bars represent s.d. $^{*}p < 0.05$, $^{**}p < 0.01$ from one-way ANOVA with Tukey HSD test. NS, no significance.

Supplementary Figure S5. Upregulation of gene expression and induction of protein levels of CXCL1 and CXCL8 in iNF-60 in the co-culture of 44As3 cells. (a) The expression of *CXCL1*, *CXCL8* and *ACTA2* in iNF-60 cells. n = 3 biological

replicates. **(b)** ELISA analysis of CXCL1 and CXCL8 secretions in the conditioned medium of iNF-60. Error bars represent s.d. *$p < 0.05$, from one-way ANOVA with Tukey's HSD test. NS, no significance.

Supplementary Figure S6. The uptake of DGC cell-derived EVs into the fibroblasts. (a) Schematic protocol for the uptake of EVs into the fibroblasts. **(b)** Representative confocal microscopic images. PKH-67 green-labeled EVs derived from HSC-44PE and 44As3 were added into iNF-58. Scale bars, 50 μm. **(c)** The percentage of PKH-67 positive cells on the fibroblasts. Ten hours after the treatment of PKH-67 labelled EVs from 44As3 cells, PKH67-positive fibroblasts were observed by fluorescence microscopy and the percentage was calculated.

Supplementary Figure S7. The effect of EVs and TGF-β on the expression of chemokines and myofibroblast-related genes. (a) The effect of EVs on the *ACTA2* and *COL4A1* expression in iNF-58 cells treated with TGF-β. $n = 3$ biological replicates. Error bars represent s.d. *$p < 0.05$, **$p < 0.01$ from one-way ANOVA with Tukey's HSD test. NS, no significance. **(b)** The effect of the EVs-depleted culture medium (As3 CM exo depl) of 44As3 cells on the induction of *CXCL8* in iNF-58. $n = 2$ biological replicates; $n = 3$ technical replicates. Error bars represent s.d. **$p < 0.01$ from one-way ANOVA with Tukey's HSD test. NS, no significance.

Supplementary Figure S8. The effect of Chemokines on the cell proliferation and invasion of 44As3. (a) MTT assay showing the number of 44As3 cells with or without recombinant CXCL1 and CXCL8 proteins at each time point. $n = 3$ biological

replicates. Error bars represent s.d. **(b)** Invasion activity of 44As3 with or without recombinant CXCL1 and CXCL8 proteins. Error bars represent s.d. Student's *t*-test. NS, no significance. $n = 3$ biological replicates.

Supplementary Figure S9. The proteome analysis and miRNA expression of high- and low-metastatic DGC cell-derived EVs. (a) The spectral counts of conventional EV markers in each EV. **(b)** GO analysis with selected proteins which were significantly enriched in the 44As3 EVs or HSC-44PE EVs. **(c)** A heat map showing 756 differentially expressed miRNAs detected in both HSC-44PE EVs (PE EVs) and 44As3 EVs (As3 EVs). $n = 3$ biological replicates. **(d)** PCA of miRNA expression of HSC-44PE EVs (PE EVs) and 44As3 EVs (As3 EVs).

Supplementary Figure S10. Decreased CXCL1 and CXCL8 expressions after inhibiting miRNAs in the high metastatic gastric cancer cell line, 44As3. (a) Experimental schedule of miRNA inhibition in the co-culture of fibroblasts with 44As3. **(b)** qRT-PCR analysis of miRNA expression after miRNA inhibition. After miR-155, miR-193b and miR-210 inhibitors were transfected, miRNA expression levels are detected by Taqman miRNA assay. NC: negative control. The average of values ($n = 2$, technical duplicate) are shown in the bar graphs. **(c)** CXCL1 and CXCL8 expression after miRNA inhibitions in 44As3 cells in the co-culture system. NC: negative control. The values are normalized with NC samples. $n = 3$, biological replicate. p value was calculated by Student's t-test. * $p < 0.05$.

Supplementary Figure S11. Chemokine genes and *ACTA2* expression were closely associated with patient poor prognosis. (a) Kaplan-Meier analysis for the probability of overall survival in the intestinal-type GC and DGC patients according to the CXCL1 expressions. **(b)** Kaplan-Meier analysis for the probability of overall survival in intestinal-type GC patients according to the *CXCL8* expressions. **(c)** Kaplan-Meier analysis for the probability of overall survival in (left) intestinal-type GC, (middle) DGC, and All type GC (right) patients according to the *ACTA2* expressions. Hazard ratios (HRs) and *p*-values (log rank test) are shown for each survival analysis. Kaplan–Meier curves were generated using the KMplot software from a database of public microarray data sets (http://kmplot.com/analysis).

Supplementary Figure S12. The relationship between CXCL8 and patient poor prognosis. (a) Representative microscopic images of CXCL8 in the malignant epithelial cells and the inflammatory cells. **(b)** Kaplan-Meier analysis for the probability of overall survival in 86 gastric cancer patients according to the positive (n = 31) or negative (n = 55) of CXCL8 in the malignant epithelial cells. **(c)** Kaplan-Meier plot for the probability of overall survival in gastric cancer patients with CXCL8 in the malignant epithelial cells only (CXCL8 in epi only, n = 12), CXCL8 in the CAFs only (CXCL8 in caf only, n = 18). **(d)** Kaplan-Meier analysis for the probability of overall survival in 86 gastric cancer patients according to the positive (n = 27) or negative (n = 59) of α-SMA expression in the CAFs.

Supplementary Figure S13. Immunohistochemistry analysis of CXCL8 and FAP for human and mouse tissue sections. Representative immunofluorescence images of

FAP (red), CXCL8 (green), and DAPI nuclear counterstaining (blue) in human gastric cancer. Scale bars, 50 μm.

Supplementary Figure S14. Schematic representation of the function of EVs derived from High- metastatic DGC cells on the induction of CAF subpopulations. High-metastatic DGC cells can create two distinct fibroblast subpopulations: a myofibroblastic phenotype and a chemokine-expressing phenotype. EVs derived from high-metastatic DGC cells contribute to the formation of chemokine-expressing fibroblasts by transferring several miRNAs. On the other hand, the myofibroblastic phenotype could be induced in an EV-independent mechanism (e.g. TGF-β signalling pathways). These subpopulations may be associated with the appropriate tumour microenvironment for gastric cancer metastasis.

Supplementary Methods

Cell cultures. The HSC-44PE and 44As3 cell lines were used as described previously [1]. Two human normal gastric fibroblasts (NFs), NF-58 and 60 were established from the “normal” non-neoplastic primary tumour site of GC tissues [2]. NF-58 was derived from diffuse-type GC patients. NF-60 was derived from patients with intestinal-type GC patients. The primary NFs were immortalized by following infection with retroviruses expressing mutant Cdk4, cyclin D1 and human telomerase reverse transcriptase: iNF-58 and iNF-60 [3]. Briefly, GC cell lines were cultured in RPMI-1640 (Thermo Fisher Scientific, Rockford, IL) supplemented with 10% heat-inactivated fetal bovine serum (FBS, Gibco, Thermo Fisher Scientific) and 1% Antibiotic-Antimycotic (Thermo Fisher Scientific) at 37 °C in 5% CO2. iNF-58 and iNF-60 were cultured in Dulbecco’s modified Eagle medium (DMEM; Thermo Fisher Scientific, Rockford, IL) with 10% FBS and 1% Antibiotic-Antimycotic (Thermo Fisher Scientific) and 0.5 mM sodium pyruvate (Sigma-Aldrich, St. Louis, MO). Cell lines were tested for mycoplasma by PCR (e-MycoTM VALiD Mycoplasma PCR Detection Kit, iNtRON, Seoul, Korea).

For trans-well co-cultures, $3x10^4$ of HSC-44PE or 44As3 cell lines were seeded into the top of trans-well membrane (0.4 μm pore size, Corning Life Science, Tewksbury, MA) with iNF-58 or iNF-60 ($3x10^4$) growing in the lower compartment in 6-well plate in DMEM (Thermo Fisher Scientific) with 10% FBS, 1% Antibiotic-Antimycotic (Thermo Fisher Scientific) and 0.5 mM sodium pyruvate (Sigma-Aldrich).

Immunofluorescence (IF) and immunohistochemistry (IHC). For tissue sections, the primary tumour tissues of the orthotopic mouse model and GC clinical tissue samples were fixed with formalin and embedded in paraffin. Following dewaxing and

rehydration, the antigen retrieval was performed by boiling the specimens in 1/200 diluted ImmunoSaver (Nissin EM, Tokyo, Japan) at 98 °C for 45 min or microwave in citrate buffer for 15 min. For IHC, after peroxidase activity was blocked with 3% H_2O_2 in methanol for 30 min, the sections were incubated with 0.1% Triton X-100 for permeabilization. After treatment with a blocking reagent (Nacalai Tesque, Kyoto, Japan) at 4 °C for 30 min, the specimens were incubated with primary antibodies, anti-α-SMA (#19245, Cell Signaling Technology, Beverly, MA), anti-CAM5.2 (#349205, BD Bioscience, San Joe, CA), anti-CXCL8 (MAB208, R&D systems, Minneapolis, MN), anti-CD206 (AF2535, R&D systems), anti-MPO (AF3667, R&D systems), anti-LYVE-1 (AF2125, R&D systems), and anti-Human mitochondria (ab92824, Abcam, Cambridge, MA), anti-Luciferase (PM016, MBL, Tokyo, Japan), and anti-FAP (AF3715, R&D systems) at room temperature for 1 h or at 4 °C overnight. And then, the following procedures of IF or IHC were performed.

For cell lines, the immortalized fibroblasts were washed with PBS (-) and 4% para-formaldehyde was then added. The fixed immortalized fibroblasts were permeabilized with 0.1% Triton-X-100 in PBS (-) for 5 min and blocked with 10% FBS in PBS (-) for 30 min before staining. Then, the samples were incubated with primary antibodies, which included anti-α-SMA (#19245, Cell Signaling Technology), anti-vimentin (#5741, Cell Signaling Technology) and anti-CXCL8 (MAB208, R&D systems) at room temperature for 60 min.

For IF, appropriate secondary antibodies linked with Alexa Fluor 488 (Molecular Probes, Eugene, OR) or Alexa Fluor 594 (Molecular Probes) were applied, followed by incubation at room temperature for 60 min. Cover slips were mounted onto the tissue sections or cells with the VECTASHIELD HardSet mounting medium with

DAPI (Vector Laboratories, Inc., Burlingame, CA) and images acquired using a BZ-X700 microscope (Keyence, Osaka, Japan) and analyzed using the image analysis application for the BZ-X700 microscope (Keyence), or using a FV10i confocal laser scanning microscope (Olympus, Tokyo, Japan).

For IHC, the tissue sections were stained using ImPRESS IgG-peroxidase kits (Vector Labs, Burlingame, CA) and a metal-enhanced DAB substrate kit (Thermo Fisher Scientific), according to the supplier's instructions. After counterstaining with haematoxylin, specimens were dehydrated and mounted. The tissue sections were also stained with hematoxylin and eosin. The images acquired using a BZ-X700 microscope (Keyence) and analyzed using the image analysis application for the BZ-X700 microscope (Keyence).

***In vivo* studies.** Animal experiments were performed in compliance with the guidelines of the Institute for Laboratory Animal Research, National Cancer Center Research Institute (Number: T17-069-C01); 6–8 weeks old female BALB/c nude mice (Charles River Laboratories Japan, Inc., Kanagawa, Japan) were used in the experiments. Mice were divided into each group randomly. A small median abdominal incision (around 1 cm) was made in the mice. The stomach was pulled out, and DGC cell lines (1×10^6 cells in 50 μl of PBS (-)) were injected into the middle wall of the greater curvature of the glandular portion of the stomach. HSC-44PE or 44As3 DGC cell lines were transplanted. For in vivo imaging, the mice were administered 150 mg kg^{-1} D-luciferin (Promega, Madison, WI) by intraperitoneal injection. Ten minutes later, photons in the whole bodies of the animals were measured by assessing bioluminescence with an IVIS Spectrum imaging system (Caliper Life Science, Hopkinton, MA). The data were

analyzed using LIVINGIMAGE 4.4 software (Caliper Life Science). Tumour development was monitored by bioluminescent imaging.

Extracellular vesicle (EV) purification and analysis. The cells were washed with PBS (-), and the culture medium was replaced with RPMI-1640 medium (Thermo Fisher Scientific) for HSC-44PE and 44As3 containing an AA (but not FBS). After incubation for 48 h, the conditioned medium (CM) was collected and centrifuged at 2,000 × g for 10 min at 4 °C. To thoroughly remove cellular debris, the supernatant was filtered through a 0.22 μm filter (Millipore, Billerica, MA). The CM of DGC cell lines was then used for EV isolation.

The EV purification was performed as described previously [4]. Briefly, CM of DGC cell lines was ultracentrifuged at 35,000 r.p.m. using a SW41Ti rotor for 70 min at 4 °C. The pellets were washed with PBS (-), ultracentrifuged at 35,000 r.p.m. using the SW41Ti rotor for 70 min at 4 °C and resuspended in PBS (-). The isolated EVs were visualized using a phase-contrast transmission electron microscope as described previously [4]. The protein concentration of the putative EV fraction was determined by a Quant-iT Protein Assay with a Qubit 2.0 Fluorometer (Thermo Fisher Scientific). To determine the size distribution of the EVs, nanoparticle tracking analysis was carried out using the Nanosight system (NanoSight Ltd, Amesbury, UK) on samples diluted 400-fold with PBS (-) for analysis.

PKH67-labelled EV transfer. EVs derived from HSC-44PE or 44As3 GC cell lines were labelled with a PKH67 green fluorescence labelling kit according to the manufacturer's instructions (Sigma-Aldrich). Briefly, purified EVs were incubated with

2 mM of PKH67 for 5 min, and then washed them using a 100 kDa filter (Microcon YM-100, Millipore) to remove excess PKH-67 dye. PKH67-labelled EVs were treated to iNF-58. After 6 - 10 hr incubation, images were acquired using a confocal microscope (FV10i; Olympus Life Science).

RNA extraction and quantitative RT-PCR (qRT-PCR). Total RNA and miRNA were extracted from cancer-derived EVs (10 μg) and cultured cells using QIAzol and the miRNeasy Mini Kit (Qiagen, Hilden, Germany) according to the manufacturer's protocols. For mRNA expression by qRT-PCR analysis, complementary DNA (cDNA) was generated from total RNA using a High Capacity cDNA Reverse Transcription Kit (Thermo Fisher Scientific). Real-time PCR was subsequently performed in triplicate with cDNA using Platinum SYBR Green qPCR SuperMix UDG (Thermo Fisher Scientific). For miRNA expression by qRT–PCR analysis, cDNA was generated from total RNA using a TaqMan microRNA Reverse Transcription Kit (Thermo Fisher Scientific). TaqMan probes were purchased from Thermo Fisher Scientific (**Supplementary Table S6**). Real-time PCR was subsequently performed in triplicate with cDNA using a Universal PCR Master Mix (Thermo Fisher Scientific). The data were collected and analyzed using a StepOne Real-Time PCR System and StepOne Software v2.3 (Thermo Fisher Scientific). All mRNA quantification data from cultured cells were normalized to the expression of β-actin (ACTB) and all primer sequences are listed in **Supplementary Table S6**.

Microarray and bioinformatics. mRNA and miRNA microarray were performed as described previously [5]. Briefly, to perform a mRNA microarray, 3×10^4 of HSC-44PE

or 44As3 were seeded into the top of trans-well membrane (0.4 μm pore size, Corning Life Science) with iNF-58 and iNF-60 ($3x10^4$) growing in the lower compartment in 6-well plate and incubated for one week. iNF-58 ($2x10^4$ cells) were prepared with HSC-44PE EVs, 44As3 EVs, PBS (-), or no treated (NT) into 24-well plates and incubated for five days. Total RNA was extracted from these cells as described above. The Agilent SurePrint G3 Human GE v3 8x60K Microarray (design ID: 072363, Agilent Technologies Inc., Santa Clara, CA) was used for this analysis and the microarray chips were scanned using an Agilent DNA microarray scanner (Agilent Technologies Inc.). Intensity values of each scanned feature were quantified using Agilent Feature Extraction software version 11.5.1.1, which performs background subtractions (Agilent Technologies Inc.). Normalization was performed with Agilent GeneSpring version 14.9 (per chip: normalization to 75th percentile shift). The altered transcripts were quantified using the comparative method. Raw and normalized microarray data are available in the Gene Expression Omnibus database (accession numbers GSE116167 and GSE116176).

To perform miRNA microarray, miRNA was extracted from 10 μg of HSC-44PE EVs or 44As3 EVs as described above. The Agilent Human miRNA 8x60K Microarray V21.0 (design ID: 70156, Agilent Technologies Inc.) was used for this analysis and the microarray chips were scanned using an Agilent DNA microarray scanner (Agilent Technologies Inc.). Intensity values of each scanned feature were quantified using Agilent Feature Extraction software version 11.5.1.1, which performs background subtractions (Agilent Technologies Inc.). Normalization was performed with Agilent GeneSpring GX version 14.8 (per chip: normalization to 75th percentile

shift). Raw and normalized microarray data are available in the Gene Expression Omnibus database (accession numbers GSE116143).

The intensity values were log2-transformed and imported into the Partek Genomics Suite 6.6 (Partek Inc., Chesterfield, MO, USA). For gene expression analysis, a one-way analysis of variance was performed to identify differentially expressed genes. *p*-values and fold-change numbers were calculated for each analysis. Unsupervised clustering and heat map generation were performed with sorted datasets by Pearson's correlation on Ward's method with selected probe sets by Partek Genomics Suite 6.6. GSEA (www.broadinstitute.org/gsea) was performed for mono-culture, co-culture and EV treatment.

Immunoblot analysis and ELISA. For immunoblot analysis, whole-cell lysates were prepared with Mammalian Protein Extract Reagent (M-PER; Thermo Fisher Scientific). The whole-cell lysates (20 μg) were solubilized in Laemmli sample buffer by boiling and then loaded onto a Mini- PROTEAN TGX Gel (4 - 15 %, Bio-Rad) and electrotransferred (100 V, 30 mA). The proteins were transferred to a polyvinylidene difluoride membrane (Millipore). After blocking in Blocking One (Nacalai Tesque), the membranes were incubated for 1 h at room temperature with primary antibodies, which included anti-CD63 (556019, dilution 1 : 200, BD Bioscience), anti-CD9 (sc-59140, dilution 1 : 200, Santa Cruz Biotechnology Inc., Santa Cruz, CA), anti-CD81 (sc-23962, dilution 1 : 200, Santa Cruz Biotechnology Inc.), anti-actin (MAB1501, dilution 1 : 1,000, Millipore), anti-HSP70 (#610607, dilution 1 : 1,000, BD Bioscience). Secondary antibodies (horseradish peroxidase-linked anti-mouse IgG, NA931 or horseradish peroxidase-linked anti-rabbit IgG, NA934, GE Healthcare, Milwaukee, WI) were used

at a dilution of 1 : 5,000. The membrane was then exposed to ImmunoStar LD (Wako, Osaka, Japan).

Quantitation of secreted CXCL1 and CXCL8 in the conditioned medium were performed using the Quantikine ELISA Kit (R&D Systems) according to the manufacturer's instructions.

Cell transfection. Transfection of cells was performed with Lipofectamine RNAiMAX Reagent (Thermo Fisher Scientific) according to the manufacturer's instructions. Briefly, the cells were seeded at 50-70 % confluence the day before transfection. For transfection of the miRNA mimics, miR-155-5p (MC12601), miR-210-3p (MC10516), miR-205-5p (MC11015), miR-192-5p (MC10456), miR-193b-3p (MC12383), miR-150-5p (MC10070), miR-7-5p (MC10047), miR-1273g-3p (MC23749), miR-378e-3p (MC11360), and miRNA mimic negative control (Ambion, Austin, TX) were used for each transfection at a final concentration of 50 nM. For transfection of the miRNA inhibitors, miR-155-5p (MH12601), miR-193b-3p (MH12383), miR-210-3p (MH10516), and miRNA inhibitor negative control (Ambion) were used at a final concentration of 20 nM. After 5 day of incubation, CXCL1 and CXCL8 gene expressions and protein levels in fibroblasts were examined by qRT-PCR and ELISA, respectively, as described above.

Cell proliferation assay. 44As3 cell line (2000 cells) were seeded into each well of a 96-well plate and recombinant CXCL1 or CXCL8 (0 ng/mL, 40 ng/mL, R&D systems) were treated. Cell viability as a measure of relative proliferation in an MTS [3-(4,5-dimethylthiazol-2-yl)-5-(3-carboxymethoxyphenyl)-2-(4-sulfophenyl)-2-(4-

sulfophenyl)-2H-tetrazolium] assay was determined on the indicated days using the Cell Counting Kit-8 (Dojindo, Kumamoto, Japan) according to the manufacturer's instructions, and the absorbance at 450 nm was measured using an Synergy™ H4 Microplate Reader (BioTek, Winooski, VT).

Quantification of microscopic images in vitro and in vivo. The quantification of the α-SMA- and CXCL8-positive fibroblasts in mono-culture or co-culture were determined by using the image analysis application software for the BZ-X700 microscope (Keyence). Briefly, fibroblasts in the culture plates were scanned at 10x objective magnification, and the areas with highest density of these markers-positive cells were identified as a "hot spots". The five "hot spots" were observed at 20x objective magnification, and then used for the quantification. The area and cell number of these markers were normalized with the number and the area of DAPI-positive cells.

The quantification of the LYVE-1-, MPO- and CD206-positive cells within tumour mass were also analyzed using the image analysis application for the BZ-X700 microscope (Keyence). The cell number and area were calculated from the positive area of these markers determined by the application software in the multiple fields (20x objective magnification). For the quantification of LYVE-1 and CD206, the positively stained area size was evaluated. The area and cell number of these markers were normalized to whole tumour area.

The extent of IHC for CXCL8 and α-SMA in human GC tissue samples was evaluated microscopically. In IHC for CXCL8 and α-SMA in CAFs, the cases with considered as positive if at least 5 fibroblasts right next to GC cells were stained in one high power field (HPF) on average. Positive fibroblasts were counted and summed from

10 HPF. For epithelial-malignant cells, when more than 10% of the cancer cells were stained, it was considered as the positive case.

Proteomic analysis. Forty μg of EV proteins for each cell line were used for the proteomic analysis. A mass spectrometric analysis was carried out using LC-MS/MS. The LC-MS/MS parameter and methods were described previously [6]. The database search was performed with MASCOT (Matrix Science, London, UK). The generated pkl files were submitted to SWISS-PROT; the search parameters were as follows: static modifications, carbamidomethyl (C); Dynamic modifications, oxidation (M); missed cleavages, up to 2; fragment ion mass, 0.8 Da; taxonomy, human. Protein identifications were validated using Scaffold (Proteome Software Inc., Portland, OR). Protein identifications were accepted if they were detected with ≥90.0% by the Scaffold local false discovery rate algorithm. Fisher's exact test p value in Scaffold were calculated according to a model previously describe [7].

Kaplan–Meier analysis. Kaplan–Meier analysis was performed using the KMplot software from a database of public microarray data sets (http://kmplot.com/ analysis) and using the R statistical software (http://www.r-project.org/) [8] from our IHC data of CXCL8 in 86 GC patients. From database, the results were collected from 572 GC patients at all types including 176 intestinal-type GC and 105 diffuse-type GC patients. Kaplan–Meier plots were generated for the *CXCL1* probe (204475_at), *CXCL8* probe (202859_x_at) and *ACTA2* probe (215787_at). To analyze the prognostic value of the probe, the samples were split into two groups according to the cutoff value generated by the software. The outlier arrays and the markedly different characteristic dataset

(GSE62254) were excluded in this analysis. HRs and *p* values (log rank P) are shown for each survival analysis.

Supplementary references

1. Yanagihara K, Tanaka H, Takigahira M, Ino Y, Yamaguchi Y, Toge T, et al. Establishment of two cell lines from human gastric scirrhous carcinoma that possess the potential to metastasize spontaneously in nude mice. Cancer Sci. 2004;95:575-82.

2. Itoh G, Chida S, Yanagihara K, Yashiro M, Aiba N, Tanaka M. Cancer-associated fibroblasts induce cancer cell apoptosis that regulates invasion mode of tumours. Oncogene. 2017;36:4434-44.

3. Sasaki R, Narisawa-Saito M, Yugawa T, Fujita M, Tashiro H, Katabuchi H, et al. Oncogenic transformation of human ovarian surface epithelial cells with defined cellular oncogenes. Carcinogenesis. 2009;30:423-31.

4. Yokoi A, Yoshioka Y, Yamamoto Y, Ishikawa M, Ikeda SI, Kato T, et al. Malignant extracellular vesicles carrying MMP1 mRNA facilitate peritoneal dissemination in ovarian cancer. Nat Commun. 2017;8:14470.

5. Tominaga N, Kosaka N, Ono M, Katsuda T, Yoshioka Y, Tamura K, et al. Brain metastatic cancer cells release microRNA-181c-containing extracellular vesicles capable of destructing blood-brain barrier. Nat Commun. 2015;6:6716.

6. Hagiwara K, Katsuda T, Gailhouste L, Kosaka N, Ochiya T. Commitment of Annexin A2 in recruitment of microRNAs into extracellular vesicles. FEBS Lett. 2015;589:4071-8.

7. Zhang B, VerBerkmoes NC, Langston MA, Uberbacher E, Hettich RL, Samatova NF. Detecting differential and correlated protein expression in label-free shotgun proteomics. J Proteome Res. 2006;5:2909-18.

8. R Core Team (2017) R: A Language and Environment for Statistical

Computing.
